# MicroRNA-93 activates c-Met/PI3K/Akt pathway activity in hepatocellular carcinoma by directly inhibiting PTEN and CDKN1A

**DOI:** 10.18632/oncotarget.3085

**Published:** 2014-12-26

**Authors:** Katsuya Ohta, Hiromitsu Hoshino, Jinhua Wang, Shigeshi Ono, Yuuki Iida, Keisuke Hata, Sharon K. Huang, Steven Colquhoun, Dave S. B. Hoon

**Affiliations:** ^1^ Department of Molecular Oncology, John Wayne Cancer Institute at Providence Saint John's Health Center, Santa Monica, CA, USA; ^2^ Liver Disease and Transplant Center, Cedars-Sinai Medical Center, Beverly Hills, CA, USA

**Keywords:** miR-93, hepatocellular carcinoma, drug-sensitivity, sorafenib and tivantinib

## Abstract

To assess the role of microRNAs (miR) in hepatocellular carcinoma (HCC), we performed comprehensive microRNA expression profiling using HCC cell lines and identified miR-93 as a novel target associated with HCC. We further verified miR-93 expression levels in advanced HCC tumors (n=47) by a direct PCR assay and found that elevated miR-93 expression level is significantly correlated with poor prognosis. Elevated miR-93 expression significantly stimulated *in vitro* cell proliferation, migration and invasion, and additionally inhibited apoptosis. We confirmed that miR-93 directly bound with the 3′ untranslated regions of the tumor-suppressor genes *PTEN* and *CDKN1A*, respectively,and inhibited their expression. As a result of this inhibition, the c-Met/PI3K/Akt pathway activity was enhanced. IHC analysis of HCC tumors showed significant correlation between c-Met protein expression levels and miR-93 expression levels. Knockdown of c-Met inhibited the activation of the c-Met/PI3K/Akt pathway regardless of hepatocyte growth factor (HGF) treatment, and furthermore reduced the expression of miR-93 in these HCC cells. miR-93 also rendered cells to be more sensitive to sorafenib and tivantinib treatment. We concluded that miR-93 stimulated cell proliferation, migration, and invasion through the oncogenic c-Met/PI3K/Akt pathway and also inhibited apoptosis by directly inhibiting *PTEN* and *CDKN1A* expression in human HCC.

## INTRODUCTION

Hepatocellular carcinoma (HCC) remains a leading cause of cancer deaths worldwide, in part because many patients are diagnosed initially with advanced disease. Currently, there are several first line options including the approved systemic therapeutic drug sorafenib [1-3]. Sorafenib is a therapeutic inhibitor of multiple tyrosine kinases, including Ras-mitogen activated protein kinase (MAPK; C-Raf and B-Raf) and vascular endothelial growth factor receptor-2 (VEGFR-2). Sorafenib can modestly prolong overall survival among previously untreated HCC patients with Child-Pugh class A liver cirrhosis [1, 2]. Another tyrosine kinase inhibitor, tivantinib, which inhibits c-Met tyrosine kinase, has been recently reported to be a promising treatment candidate for advanced HCC [4].

HCC carcinogenesis is related to inflammation events [5, 6]. c-Met, a receptor for hepatocyte growth factor (HGF), which is a promoter of cancer progression, has several major alternative downstream pathways in its signaling cascade: Akt 1, 2 and 3 (protein kinase B), signal transducer and activator of transcription 3 (STAT3), and MAPK. Studies of the Akt pathway have shown that a chromosome 10 deletion of phosphoinositide 3-kinase (PI3K)/Tensin homology protein (PTEN) can facilitate tumorigenesis [7-9]. *PTEN* is a plasma membrane lipid phosphatase and tumor suppressor that dephosphorylates phosphatidylinositol 3,4,5-triphosphate (PIP3) back to biphosphate (PIP2), thereby inhibiting the phosphorylation of Akt [10, 11]. The theranostic benefit of targeting this pathway has been demonstrated in several types of cancers [12].

MicroRNAs (miRs) have been shown to be important post-transcriptional regulators of gene expression in cancer cells as well as normal cells. These noncoding small RNAs bind to 3′ untranslated regions (3′UTRs) of mRNA of specific genes [13, 14]. miRs have the ability to significantly modulate gene expression [15]; therefore, assessment of miR levels could potentially be used for the classification and stratification of tumors [13, 14, 16-18]. Specific miRs, such as miR-30a, 122, and 148a, have been demonstrated to play a role in the development of physiological function in normal liver [19-21]. The clinical importance of miRs in cancer progression, especially in regards to response to chemotherapy, has also been demonstrated [22, 23].

In this study, we evaluated the miR expression profiles of HCC and non-HCC cell lines using miR q-PCR array analysis and identified miR-93 as a significant miR associated with HCC progression. We demonstrated that miR-93 promoted HCC cell proliferation, migration and invasion through activation of the oncogenic c-Met/PI3K/Akt pathway, and also inhibited apoptosis and drug-sensitivity by directly inhibiting *PTEN* and *CDKN1A* expression in HCC cells.

## RESULTS

### miR-93 expression is enhanced in HCC tumors

**Figure 1:**
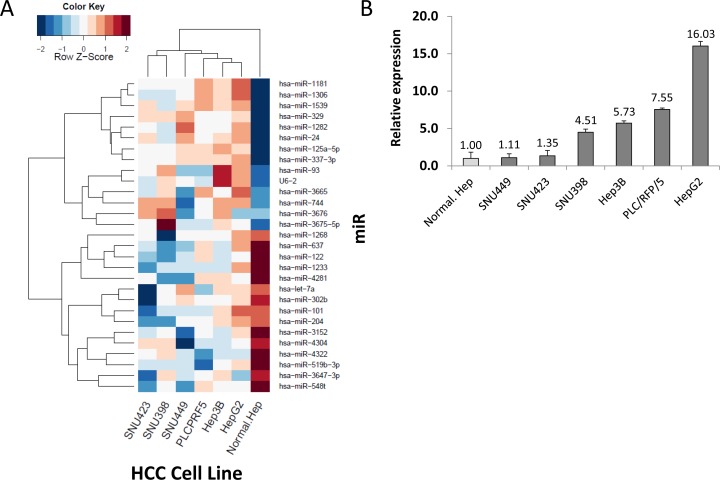
Identification of miR candidates in HCC (A) Heat map of miR sequences used to identify miRs whose relative expression levels changed by a factor of 10-fold based on a logarithmic scale. (B) Verification of miR-93 in six HCC cell lines and a normal hepatocyte cell line referenced by miR-181a.

**Figure 2:**
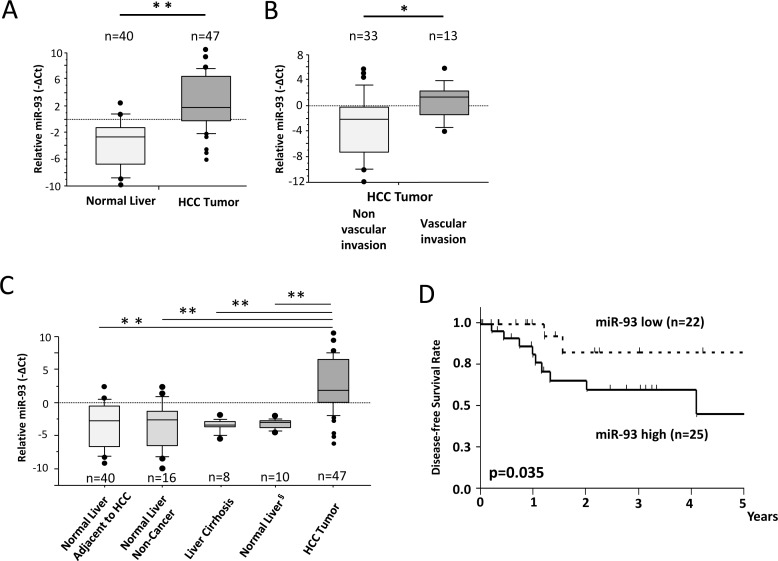
miR-93 is enhanced in HCC tumor tissues assessed in PEAT HCC clinical specimens and non-cancer liver specimens were assessed to confirm the significance of miR-93. (A) Expression of miR-93 in HCC tumors (n=47) and adjacent non-HCC tissues (n=40). (B) Expression of miR-93 was significantly higher in HCC tumors (n=13) with vascular invasion versus HCC tumors without vascular invasion (n=33). (C) Expression of miR-93 in normal liver autopsy tissues (n=16), cirrhotic liver tissues (n=8), and normal tumor-free liver tissue removed during resection of hepatic melanoma metastases (n=10). (D) Comparison of high and low expressing miR-93 in a disease-free survival curve using Kaplan Meier methods (Log-rank test, p=0.035). * p< 0.05, * *p< 0.01.

miR q-PCR array screening of six HCC cell lines identified 29 miRs whose expression levels changed more than 2-fold up or down (Figure 1A). Two candidate miRs (miR-93, miR-125a-5p) exhibited greater than a 10-fold increase in expression. We confirmed the significance of both miRs in six HCC cells compared to normal hepatocyte cells by miR PCR assays (Figure 1B and Supplemental Figure 1A). The expression levels of miR-93 and miR-125a-5p were stimulated 4.5-fold and 9-fold, respectively, in a cohort of 47 HCC tumors compared to 40 normal liver and liver cirrhosis tissues (Figure 2A and Supplemental Figure 1B). Based on the status of HCC vascular invasiveness, which is correlated to tumor progression, HCC specimens were further categorized into two groups, nonvascular invasive (n=33) or vascular invasive (n=13), for a comparison of miR expression. One specimen without information of vascular invasion was excluded from the analysis. The expression of miR-93 was significantly higher in the specimens with vascular invasion (p=0.022), while miR-125a-5p was not significantly associated with the vascular-invasive status (p=0.073) (Figure 2B and Supplemental Figure 1C). We then verified that miR-93 expression was significantly higher in HCC tumors (n=47) than normal liver tissues obtained from cancer-free patients (n=16), liver tissues from non-cancer patients with liver cirrhosis (n=8), and specimens of histopathologically tumor-free liver tissue removed during liver resection of melanoma hepatic metastases (n=10) (Figure 2C). The 47 HCC specimens were then categorized into a miR-93 high group (–ΔCq>1.4, n=25) and a miR-93 low group (–ΔCq<1.4, n=22) (Table 1). Overall survival rate and disease-free survival rate analyses were performed on these two groups. miR-93 high group was significantly correlated with worse disease-free survival (p=0.035) but not overall survival (p=0.179) (Figure 2D and Supplemental Figure 2). These results suggest that the expression of miR-93 is not only significantly higher in HCC than normal hepatocytes, but also correlates with worse disease-free survival in advanced HCC patients. Therefore, we proceeded to focus on the mechanistic action of miR-93 in HCC.

**Table 1:** Comparison of baseline characteristics of HCC specimens

	miR-93 high (n=25)	miR-93 low (n=22)	*p*-value
miR-93 (−ΔCt)			p<0.0001
[median (range)]	6.47	−0.35	
	(1.49-9.38)	(−6.38, 1.31)		
Gender			p=0.539
M	18	14	
F	7	8	
Age (yrs)			p=0.031
[median (range)]	57.5	67.3	
	(43.2-81.2)	(36.6-91.9)		
Disease			p=0.232
HBV	3	0	
HCV	15	14	
Other	7	8	
Alcohol history			p=0.778
Yes	8	9	
No	17	13	
Depth of tumor			p=0.160
pT1	2	2	
pT2	14	18	
pT3	7	1	
pT4	2	1	
Vascular invasion			p=0.088
Yes	10	3	
No	15	18	
n/s	0	1	
Differentiation			p=0.855
well	3	4	
well/mod	3	3	
mod	12	8	
mod/poor	3	3	
poor	4	4	
c-Met Score			p=0.004
3+	11	1	
2+	10	7	
1+	3	11	
0	0	2	
n/s	1	1	
Percentage of 2+ or 3+ cells			p=0.145
c-Met positive >50%	12	6	
c-Met positive <50%	13	16	

### miR-93 increases proliferation, migration and invasion of HCC cells

**Figure 3:**
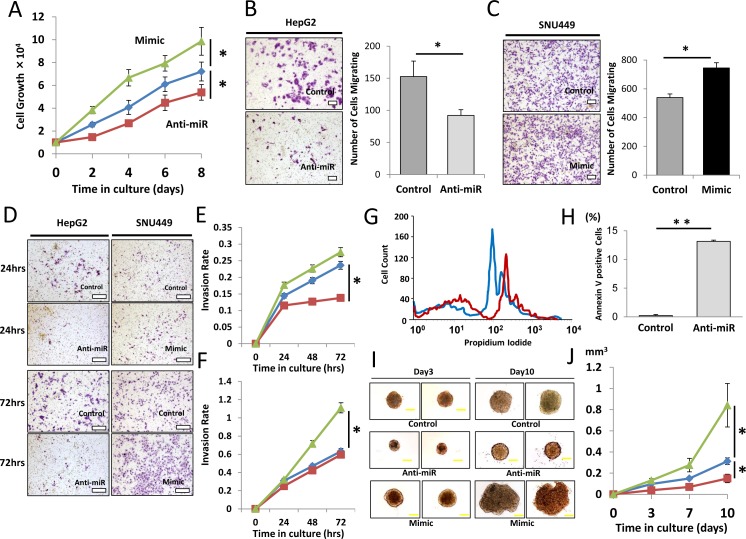
Effect of miR-93 knockdown on HCC cell function *in vitro* Clinical HCC specimens showed the relevance of miR-93, therefore we confirmed the significance *in vitro.* (A) Cell proliferation assay comparing anti-miR-93 (red), mimic-miR-93 (green) and a control-miR (blue) for 8 days. (B) Cell migration assay with anti-miR and control-miR in HepG2 cells for 24 hrs. Scale bar = 200μm. (C) Cell migration assay with mimic-miR and control-miR in SNU449 cells for 24 hrs. Scale bar = 200μm. (D) Invasion assays comparing anti-miR-93, mimic-miR-93, and control-miR. Scale bar = 300μm. (E) Invasion rate of HepG2 cells for 72 hrs. (F) Invasion rate of SNU449 cells comparing anti-miR-93 (red), mimic-miR-93 (green) and a control-miR (blue). (G) Apoptosis status monitored by propidium iodide (PI) showing a difference between control (blue) and anti-miR-93 (red) exposed cells. (H) Annexin V assay comparing HCC specimens with mock transfected cells. (I) 10 day incubation in a 3D culture with anti-miR-93, mimic-miR-93, and control-miR. Scale bar = 100μm. (J) Growth curve of tumorigenicity after treatment with anti-miR-93 and mimic-miR-93. * p< 0.05, * *p< 0.01.

To assess the role of miR-93 in HCC tumorigenesis, we performed cell proliferation, migration, invasion, and apoptosis assays in HCC cells transfected with anti-miR-93, mimic-miR-93 or control-miR. HepG2 cells, which expressed high levels of miR-93, and SNU449 cells, which expressed low levels of miR-93, were selected for the studies. Anti-miR-93 suppressed miR-93 expression 6.3-fold in anti-miR-93 transfected HepG2 cells compared to control cells, and mimic-miR-93 induced miR-93 expression 3.3-fold in mimic-miR-93 transfected SNU449 cells compared to control-miR transfected cells (Supplemental Figure 3A-B). Anti-miR-93 suppressed proliferation in HepG2 (Figure 3A). In contrast, mimic-miR-93 increased proliferation of HepG2 and SNU449 cells (Figure 3A and Supplemental Figure 4A). In addition, the migration of anti-miR-93 transfected HepG2 cells significantly decreased, whereas the migration of mimic-miR-93 transfected SNU449 cells significantly increased compared to the respective controls (Figure 3B-C and Supplemental Figure 4B-C). Similarly, the invasion rate of anti-miR-93 transfected HepG2 cells significantly decreased, whereas the invasion rate of mimic-miR-93 transfected SNU449 cells significantly increased compared to the respective controls over a 72 hour (hr) period (Figure 3D-F). To examine miR-93's effect on apoptosis, we performed flow cytometric assays to monitor the apoptosis status in HCC cells. The dead cells were visualized with propidium iodide (*PI*); anti-miR-93 transfected HepG2 cells were strongly *PI* stained relative to controls, demonstrating that apoptosis is enhanced with a loss of miR-93 function (Figure 3G). Annexin V assays were performed comparing HCC cells with mock transfected controls. The results showed 0.2% apoptosis in the control cells compared to 13.2% in anti-miR transfected cells, which also demonstrated that loss of miR-93 function by anti-miR-93 enhanced apoptosis of HCC cells (Figure 3H). In addition, we performed 3D culture analysis, which closely simulates conditions of *in vivo* tumor growth. Transfected HepG2 cells with control-, mimic-, or anti-miR-93 were grown for 10 days in regular medium condition on 3D cultured spheres (Figure 3I). Transfection of anti-miR-93 inhibited cell growth activity, while mimic-miR-93 stimulates cell growth in HepG2 cell spheres (Figure 3J).

### miR-93 inhibits *PTEN* and *CDKN1A*

**Figure 4:**
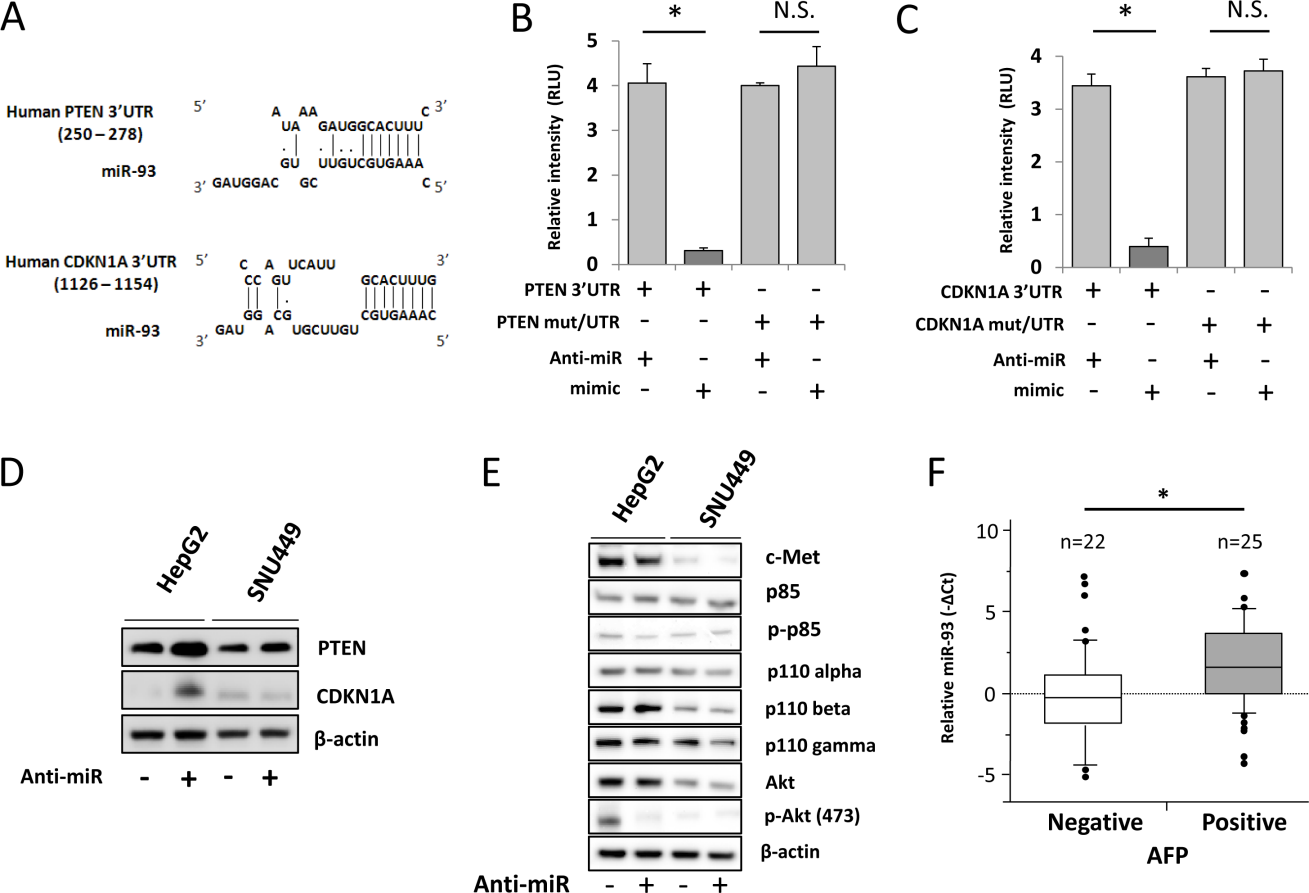
miR-93 targets PTEN and CDKN1A in HCC Pathway analysis to confirm the target of miR-93. (A) Tumor-related genes were selected using a DIANA microT v3.0 algorithm. The database showed that the potential targets of miR-93 are the *PTEN* and *CDKN1A* 3′UTR. (B) Luciferase activity in the *PTEN*-3′UTR was significantly higher in anti-miR-93 transfected HepG2 cells versus control transfected or mimic-miR-93 transfected cells. (C) *CDKN1A*-3′UTR luciferase activity. (D) Western blot analysis of protein levels of PTEN and CDKN1A upon treatment with anti-miR for 48 hrs referenced by β-actin. (E) c-Met/PI3K/Akt pathway analysis in HepG2 and SNU449. (F) Biostatistical analysis of miR-93 expression in 47 HCC tumor specimens with *AFP* mRNA status positive expression group (n=25) and negative expression group (n=22). Statistical analysis showed stimulation of miR-93 in the AFP positive group. p=0.048, Wilcoxon test. * p< 0.05.

The mechanistic effects of miR-93 on oncogenic pathways were further examined by *in-silico* analysis using the DIANA microT v3.0 algorithm database. Analysis identified *PTEN* and *CDKN1A* as candidate targets of miR-93 (Figure 4A). To demonstrate direct binding of miR-93 to these genes, we performed the co-transfection of mimic-, anti-miR-93, or control-miR with the vectors of luc-3′UTR *PTEN* or *CDKN1A* in HCC cells followed by luciferase activity analysis. To show the specificity of binding, we also performed the co-transfection of mimic- or anti-miR-93 with the vectors of luc-mut/3′UTR *PTEN* or *CDKN1A* that contained mutated sequences at the miR-93 binding site (Supplemental Figure 5). HCC cells transfected with anti-miR-93 demonstrated a 4.1-fold increase of PTEN and 3.4-fold increase of CDKN1A luciferase activity compared with HCC cells transfected with control-miR. This suggestes that *PTEN* and *CDKN1A* expression was suppressed by miR-93 binding to the 3′UTRs of those genes (Figure 4B-C). The binding was specific because the Luc-reporter vector with mutated *PTEN* and *CDKN1A* 3′UTR was not affected by the changes of miR-93 expression (Figure 4B-C). These findings were corroborated by the stimulated expression of the PTEN and CDKN1A proteins after anti-miR-93 transfection (Figure 4D). In order to understand the role of miR-93 on the c-Met/PI3K/PTEN/Akt pathway, we then analyzed the protein expressions of PI3K class 1A and 1B (p85, phospho-p85, p110-alpha, -beta, and -gamma) by western blotting of HCC cells transfected with anti-miR-93. There was no significant difference in their expression between anti-miR-93 and control-miR. Furthermore, we checked the expression of Akt and found that phospho-Akt levels were decreased by inhibiting miR-93 expression (Figure 4E).

To determine the relative expression of miR-93 and related pathways, we performed RNA sequencing on HCC cells transfected with anti-miR-93 versus control-miR transfected HCC cells. The results demonstrated the down regulation of multiple cancer-related genes (Table 2). Since α-fetoprotein (AFP) is an important HCC-specific diagnostic biomarker, we examined the association between miR-93 and *AFP* expression [24]. The expression of *AFP* was reduced by 1.6-fold in anti-miR-93 transfected cells compared to control-miR transfected cells. miR-93 did not directly inhibit the expression of *AFP* through direct binding on the 3′UTR as assessed by the DIANA microT v3.0 algorithm database, Target Scan and RNA22. Using qRT-PCR analysis, *AFP* mRNA expression was significantly lower in anti-miR-93 transfected cells than in control-miR transfected cells (Supplemental Figure 6). HCC tumor specimens (n=47) were separated into *AFP* mRNA positive (n=25) and negative groups (n=22); miR-93 expression was significantly higher in the AFP positive group compared to the negative group (p=0.048, student t-test, Figure 4F). These results suggested that miR-93 levels indirectly affect AFP activity.

**Table 2:** Relative genes in miR-93 pathway Cancerrelated genes from the RNA sequencing results. Genes were functionally categorized by apoptosis, cell growth, and drug-sensitivity.

Function	Genes
Apoptosis	*PGGT1B, PPM1D, SHF, CEL, BCL2L2. HIST1H1E, H2AFY*
Cell Growth	*NEK3, TP53RK, UHRF1, GTPBP3, SDC1, FSCN1, LLGL1. DEF6, ASB16, CRLF1, ABCD1, HIC2, DHRS2*
Drug-Sensitivity	*PBFKB4, MAD1L1, NHEJ1, RNASET2, MRPL3-18-30-40, TRIB3, TXNRD2, FOXA2, HNRNPC*

### miR-93 expression is correlated with c-Met expression

**Figure 5:**
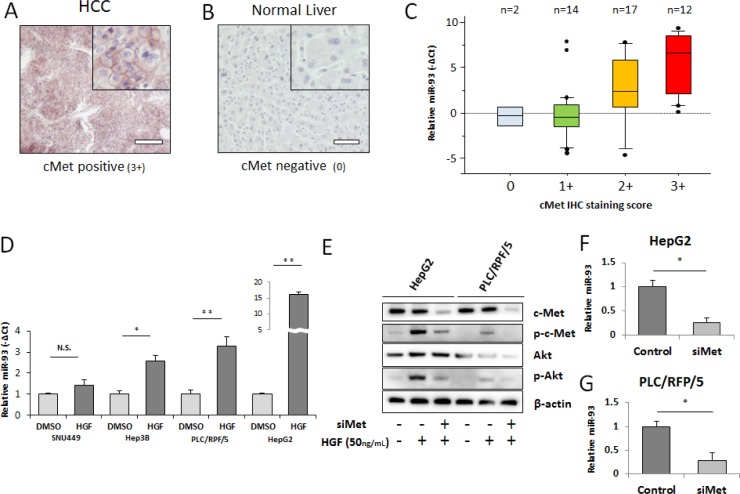
miR-93 expression correlates with HGF and c-Met IHC intensity We performed c-Met IHC staining in PEAT and examined the relationship with miR-93 expression. (A) Photomicrographs of 3-μm liver tissue sections showing strong (3+) staining for c-Met in HCC tissue and (B) negative (0) staining for c-Met in non-HCC tissue. Scale bar = 100μm. (C) Expression of miR-93 in 45 HCC tumor specimens with c-Met IHC staining status ranging from 0 to 3+. Biostatistical analysis showed significant increasing of miR-93 going from the c-Met negative group (0) to the c-Met staining group (1+, 2+ and 3+). p=0.002, Wilcoxon test. (D) Assessment of *in vitro* overexpression of miR-93 using HGF (50 ng/mL) administered for 24 hrs to 4 HCC cell lines (SNU449, Hep3B, PLC/RPF/5, and HepG2) to monitor miR-93 expression. (E) Akt pathway activity in cells with siMet and HGF treatments versus controls. (F-G) Expression of miR-93 in siMet transfected cells. * p< 0.05, * *p< 0.01.

Emerging evidence indicates that tivantinib, a c-Met inhibitor, improves median time to progression; patients with higher c-Met (3+, 2+ IHC staining in at least 50% of tumor cells) are known to have a longer time to progression with tivantinib treatment [4, 25]. To explore the relation between miR-93 and c-Met, we assessed the association of miR-93 and c-Met expression in HCC and non-HCC specimens. IHC staining of the c-Met protein expression was categorized as positive (3+, strong; 2+, intermediate; 1+, weak) or negative (0) (Supplemental Figure 7A-D). Whereas 43 of 45 (95%) HCC specimens were c-Met positive (3+, 2+, or 1+; Figure 5A), all 14 non-HCC specimens were weakly positive (1+) or negative (0) (Figure 5B). A significant correlation between c-Met IHC staining levels and miR-93 expression levels was observed (p=0.002, Wilcoxon test, Figure 5C). The relative expression of miR-93 was linearly correlated with the c-Met IHC staining score (Fisher exact test; Supplemental Figure 8). Furthermore, the percentage of positive cells (>50% or <50%) had no significant correlation with miR-93 expression. When we analyzed other cut-off points (20-80%), the miR-93 expression did not have a significant correlation with the cell number percentage of positive staining (Table 1). We focused on hepatocyte growth factor (HGF) stimulation, the ligand of c-Met, which induces the c-Met/PI3K/Akt pathway in HCC. Treatment of cell lines with 50 ng/mL HGF for 24 hrs induced the expression of miR-93; HepG2 cells were the most influenced by HGF treatment (Figure 5D). c-Met expression was suppressed by c-Met siRNA (siMet), which also suppressed phosphorylated-Akt regardless of HGF treatment (Figure 5E). The expression of miR-93 was also suppressed by siMet in HepG2 and PLC/RPF/5 cells (Figure 5F-G).

### miR-93 inhibits drug-sensitivity

**Figure 6:**
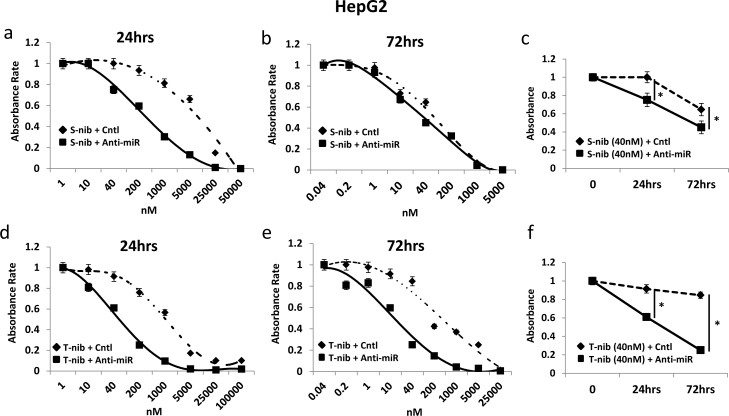

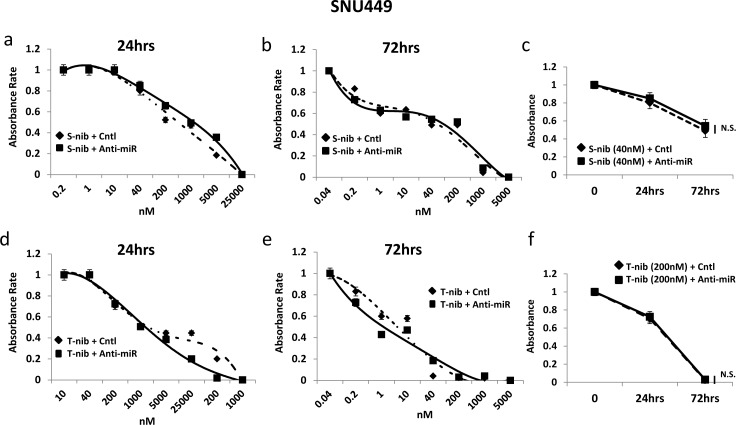
Anti-miR-93 enhanced drug-sensitivity to tyrosine kinase inhibitors Growth inhibition curves for high (HepG2) and low (SNU449) miR-93 expressing HCC cell lines in the presence of tyrosine kinase inhibitors for 24-72 hrs. (A-a) Results of anti-miR-93 and control-miR transfected HepG2 cell lines exposed to sorafenib for 24 hrs. (A-b) HepG2 cells exposed to sorafenib for 72 hrs. (A-c) Cell viability curve for HepG2 cells exposed to 40nM of sorafenib for 24-72 hrs. (A-d) HepG2 cells exposed to tivantinib for 24 hrs. (A-e) HepG2 cells exposed to tivantinib for 72 hrs. (A-f) Cell viability curve for HepG2 cells exposed to 40nM of tivantinib for 24-72 hrs. (B-a, b) SNU449 cells after transfection of anti-miR-93 in sorafenib for 24hrs and 72hrs, compared with control-miR. (B-c) Cell viability curve for SNU449 cells exposed to 40nM of sorafenib for 24-72 hrs. (B-d, e) SNU449 cells after transfection in tivantinib for 24hrs and 72hrs. (B-f) Cell viability curve for SNU449 cells exposed to 200nM of tivantinib for 24-72 hrs. * p< 0.05.

To test the sensitivity of HCC cells to specific drugs used in current treatments, we examined the response of high (HepG2) and low miR-93 (SNU449) expressing cells in the presence of two known HCC tyrosine kinase inhibitors, tivantinib and sorafenib. The combination of anti-miR-93 transfection and treatment with individual tyrosine kinase inhibitors for 24 hrs and 72 hrs significantly increased the sensitivity of HCC cells to both sorafenib (Figure 6A-a, b, c) and tivantinib (Figure 6A-d, e, f). The sensitivity was highest with the combination of tivantinib and anti-miR-93 in HepG2 cells (Figure 6A-f). The transfection of anti-miR-93 in SNU449 cells, a low miR-93 expressing cell line, had no effect on the sensitivity to both sorafenib (Figure 6B-a, b, c) or tivantinib after 24 hr and 72 hr treatment (Figure 6B-d, e, f). The combination of anti-miR-93 transfection and treatment with a tyrosine kinase inhibitor increased the sensitivity of HCC cells to tivantinib by 22.1-fold (IC50; control 1.44 ± 0.07 μM anti-miR 0.07± 0.02 μM) and sorafenib by 24.5-fold (IC50; control 8.31 ± 0.41 μM anti-miR 0.34 ± 0.02 μM) after 24 hrs, respectively. The sensitivity of HCC cells increased with tivantinib treatment by 10.1-fold (IC50; control 0.15 ± 0.07 μM anti-miR 0.02 ± 0.01 μM), and with sorafenib treatment by 2.8-fold (IC50; control 0.08 ± 0.04 μM anti-miR 0.03 ± 0.01 μM) after 72 hrs, respectively. We also examined the response of anti-miR-93 transfected HCC cells and control-miR transfected cells to chemotherapeutic agents used to treat advanced HCC patients, such as hepatic arterial infusion chemotherapy (HAIC) and transarterial chemoembolization (TACE) [26]. Cell viability assays using 5-fluorouracil (5-FU, IC50 for 24 hrs; control 27.80 ± 1.39 μM anti-miR 4.73 ± 0.23 μM, IC50 for 72 hrs; control 3.64 ± 0.18 μM anti-miR 2.26 ± 0.11μM) and cisplatin (IC50 for 24 hrs; control 46.62 ± 2.33 μM anti-miR 7.08 ± 0.35 μM, IC50 for 72 hrs; control 44.09 ± 0.02 μM anti-miR 16.53 ± 0.82 μM) showed a significant increase in the drug-sensitivity of the cell lines exposed to anti-miR-93 for 24-72 hrs (Supplemental Figure 9), suggesting that elevated miR-93 also enhances cell resistance to 5-FU and cisplatin.

**Figure 7:**
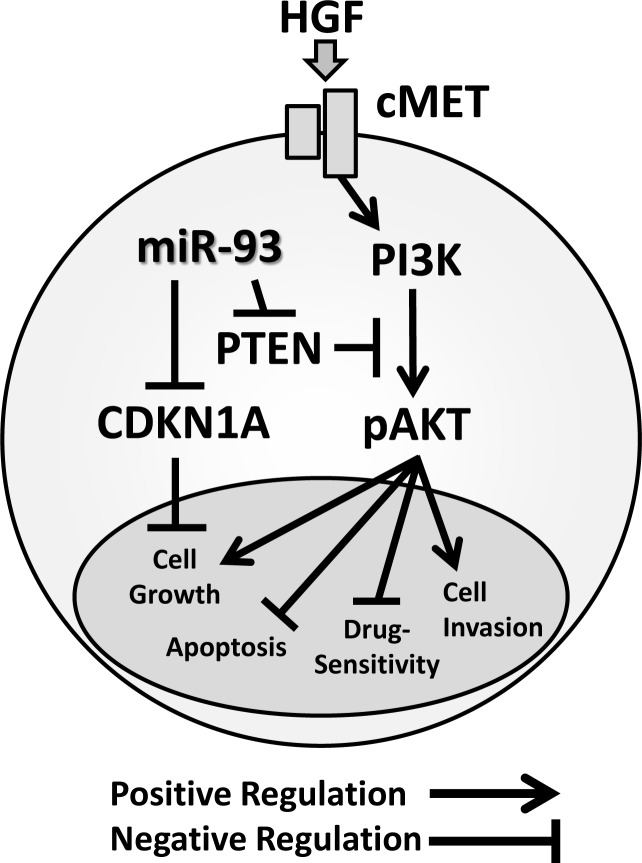
Schematic representation of miR-93 with the oncogenic c-Met/PI3K/Akt pathway miR-93 directly binds to the *PTEN* and *CDKN1A* 3′UTRs

We demonstrated that elevated miR-93 promoted HCC tumorigenesis and resistance to targeted therapy and chemotherapy (Figure 7). Overall, we also demonstrated that miR-93 inhibits the *PTEN* and *CDKN1A* genes, thereby controlling HCC cell apoptosis and c-Met/PI3K/Akt pathway activity.

## DISCUSSION

This study identified miR-93 as a novel oncogenic miR that is stimulated in both *in vitro* and *in vivo* studies in HCC. We demonstrated the mechanisms through which miR-93 inhibits *PTEN* and *CDKN1A*, thereby activating proliferation through the c-Met/PI3K/Akt pathway and inhibiting apoptosis in HCC. Previous studies have indicated that miR-93 may promote cell growth in ovarian cancer by directly targeting *PTEN* [27]. Activation of miR-93 blocked PTEN, therefore Akt phosphorylation occurred. We propose that miR-93 has an important function as a modulator of *PTEN* activity in HCC. The cyclin-dependent kinase inhibitor, *CDKN1A*, is an important factor in cell cycle regulation. Our results indicate that miR-93 inhibits both *PTEN* and *CDKN1A*. In our *in vitro* studies, we demonstrated that overexpression of miR-93 promoted cell proliferation, migration, invasion, and 3D culture sphere formation, while knockdown of miR-93 inhibited these respective functions. These results indicated that miR-93 functions as an oncogenic miR in HCC cells, however the efficacy of mimic or anti-miR-93 relies on the base miR-93 expression levels in the cells. We also demonstrated that miR-93 plays a crucial role in modulating c-Met/PI3K/Akt pathway activity. Phospho-Akt level was reduced by inhibiting miR-93 expression. This was consistent with our *hypothesis* that miR-93 induces phosphorylation of Akt through PTEN degradation. Furthermore, we demonstrated that high levels of miR-93 in tumors are significantly related to worse disease-free survival in HCC patients.

Despite the availability of different drug treatments for HCC, such as sorafenib and tivantinib, the prognosis for patients with unresectable HCC is extremely poor, with a median survival measured only in months [2]. We have demonstrated that miR-93 is associated with drug-sensitivity to 5FU, CDDP, sorafenib, and tivantinib. We also found that the inhibition of miR-93 improves the anti-HCC action of tyrosine kinase inhibitors by targeting the c-Met/PI3K/Akt pathway. To our knowledge, this is the first demonstration of its potential role in co-regulation with tyrosine kinase inhibitors in HCC.

Sorafenib, an FDA approved drug for advanced HCC patients, is an inhibitor of multiple tyrosine kinases. It provides an overall survival of 10.7 months in previously untreated HCC patients [1, 2]. Tyrosine kinase receptor pathways induce MAPK and PI3K/Akt kinase signaling in over 50% of HCC [26]. Unfortunately other therapeutic agents targeting *PI3K*, *AKT*, or *PTEN*, including new anti-angiogenic drugs, have failed to significantly improve clinical outcome in patients with HCC [28]. Sorafenib targets MAPK (C-Raf and B-Raf) and VEGFR-2, but not the PI3K/Akt pathway, which allowed us to observe the effects of miR-93 on the c-Met pathway. miR inhibits the tumor suppressor gene *PTEN* which subsequently phosphorylates *AKT* via PI3K/Akt signaling, contributing to tumorigenesis. Therefore, transfection with anti-miR-93 increases cell sensitivity to sorafenib, since inhibition of miR-93 eliminates the inhibition of *PTEN* and *CDKN1A,* thereby activates these respective genes.

A randomized phase II study indicated that tivantinib, a c-Met inhibitor, improves median time to progression; patients with higher c-Met (3+, 2+ IHC staining in at least 50% of tumor cells) reportedly had a longer time to progression with tivantinib treatment [4, 25]. Because HCC tumors with stronger c-Met staining showed higher miR-93 expression, we *hypothesized* that the efficacy of tivantinib could be associated with miR-93 expression. Interestingly, tivantinib plus anti-miR-93 was significantly more effective than sorafenib plus anti-miR-93. This study indicated that miR-93 is a novel regulator of the oncogenic c-Met/PI3K/Akt pathway in HCC, and inhibition of miR-93 hinders tumorigenesis and enhances sensitivity to conventional chemotherapy and tyrosine kinase inhibitor treatment. This is particularly important in patients with HCC since the vast majority of tumors are associated with underlying cirrhotic liver disease. Previous clinical trials using sorafenib and tivantinib were not aimed for HCC patients with Childs-Pugh B or C [28, 29]. Anti-miR-93 could improve the effectiveness of current chemotherapeutic agents and possibly decrease the dosage of those drugs, when accompanied with anti-miR-93, even for those with Childs-Pugh B or C liver disease.

The expression of AFP decreased significantly after the transfection of HCC cells with anti-miR-93, as shown by RNA sequencing data. AFP is an abundant oncoembryonal protein expressed during fetal development, but is transcriptionally repressed shortly after birth and is reactivated in HCC cells [30]. Serum AFP is a well-known diagnostic biomarker for HCC [24], as well as a useful prognostic indicator for HCC patients at the time of tumor diagnosis [31]. Patients with negative AFP have a low occurrence of tumor vascular invasion and tend to exhibit healthier hepatic function.

## CONCLUSION

We discovered miR-93 is an oncogenic miR that stimulates tumorigenesis and is a potential therapeutic target in HCC. miR-93 inhibited the *PTEN* and *CDKN1A* genes, thereby controlling HCC cell apoptosis and c-Met/PI3K/Akt pathway activity. We propose that anti-miR-93 could be used in combination with sorafenib and tivantinib to increase their respective drug-sensitivity in HCC.

## METHODS

### HCC and non-HCC liver tissues

All tissue specimens for this study were obtained according to the protocol guidelines set forth by the John Wayne Cancer Institute (JWCI) and approved by the Western Institutional Review Board (WIRB). Paraffin-embedded archival tissue (PEAT) specimens were obtained from a cohort of 47 patients diagnosed with AJCC stage I-IV HCC with paired cancer-free liver tissue adjacent to the HCC tumor (non-HCC), and 34 normal liver tissue specimens that include those from liver cirrhosis patients and normal cancer-free liver tissues adjacent to the melanoma tumor in metastatic melanoma patients.

### Cell lines

Six established HCC cell lines were obtained from the ATCC (Manassas, VA): HepG2, Hep3B, PLC/PRF/5, SNU398, SNU423, and SNU449. HepG2, Hep3B, and PLC/PRF/5 were cultured in Eagle's minimal essential medium (EMEM) and were supplemented with 10% (v/v) heat-inactivated fetal bovine serum (FBS, Gemini Bio-Products, Sacramento, CA) and antibiotics (100 U/ml penicillin and 100 μg/ml streptomycin). SNU449 and SNU423 were cultured in RPMI 1640, and supplemented with 10% (v/v) FBS and antibiotics. One normal human hepatocyte cell line (CryoHep) was obtained from BD Biosciences (Franklin Lakes, NJ). CryoHep cells were cultured in ISOM's medium supplemented with 10% FBS [32]. All cell lines were cultured in a humidified atmosphere of 5% CO_2_ at 37°C. Recombinant human HGF (R&D systems, Inc., Minneapolis, MN) was assessed at 50 ng/mL for 24 hrs in conditioned medium.

### miR qPCR array analysis

The miR microarray contained a total of 1,034 miR probes using the Human Whole Genome miRNA Profiling Kit Version 16 (ABM, Richmond, Canada). RNA was extracted and isolated from HCC cell lines (HepG2, Hep3B, PLC/PRF/5, SNU398, SNU423, and SNU449) and from CryoHep, which served as a negative control. cDNA synthesis was performed using the cDNA PolyA Tailing Reaction (Ambion, Austin, TX), and miRNA EasyScript^TM^ cDNA Synthesis Kit (ABM). Each sample was assessed with real-time instrumentation on the LightCycler480 (Roche, Indianapolis, IN) detecting the SYBR green fluorescence. Results were analyzed with the software provided by ABM. Fold changes and p-values were used to filter the differentially expressed miRs.

### miR interference (anti-miR) and mimic miRs (mimic-miR)

2×10^5^ HCC cells were transfected with 36 nM of mirVana human miRNA inhibitor (anti-miR-93) or with negative-control (control-miR) oligonucleotides (Ambion). Mimic-miR transfection was performed using 36 nM of miRIDIAN microRNA mimic-miR and negative-control (control-miR) oligonucleotides (Thermo Scientific, Waltham, MA). c-Met silencing was performed by transfection using 10 nM of Trilencer-27 siRNA (Origene, Rockville, MD). Oligonucleotides were transfected into subconfluent cells using Lipofectamine RNAiMAX for 48 hrs (Invitrogen-Life Technologies, Carlsbad, CA).

### Luminescent reporter gene transfections and luciferase assays

The luciferase reporter system that was used included an optimized Renilla luminescent reporter gene (RenSP), a genome-wide collection of promoters and the 3′UTR GoClone reporter constructs [33]. For the transfection assay, the HepG2, Hep3B, and PLC/PRF/5 cells were transfected with 200 ng of *PTEN*, [33]. For the transfection assay, the HepG2, Hep3B, and PLC/PRF/5 cells were transfected with 200ng of *PTEN*, *CDKN1A*, *PTEN-mut* and *CDKN1A-mut* 3′UTR GoClone reporter constructs using an experimental luciferase RenSP control plasmid (Switch Gear Genomics, Menlo Park, CA).

### Drug-sensitivity assay

We examined the relationship between miR-93 expression and cell sensitivity to two chemotherapeutic drugs for advanced HCC (5-FU and cisplatin) and two tyrosine kinase inhibitors. Three HCC cell lines (HepG2, Hep3B, and PLC/RFP/5) were treated with either anti-miR-93 or control-miR and were divided into 96-well micro plates. To analyze the sensitivity to 5-FU and cisplatin, cells (1×10^3^) were treated with 0 to 2mM 5-FU (Sigma-Aldrich, St. Louis, MO) or 0 to 1 mM, cisplatin (Santa Cruz Biotechnology, Inc., Dallas, TX). To analyze the sensitivity to sorafenib (Selleck Chemicals, Houston, TX) and tivantinib (Selleck Chemicals), cells (5×10^3^) were treated with 0 to 50 μM sorafenib or 0 to 25 μM tivantinib. After 24 or 72 hrs, cell viability was determined by the CellTiter-Glo Luminescent Cell Viability Assay Kit (Promega, Fitchburg, WI). All assays were assessed in triplicate.

### Proliferation, migration, invasion and 3D culture assays

Cell proliferation was quantified by measurements of bromodeoxyuridine (BrdU) incorporation during DNA synthesis in replicating (cycling) cells, using the BrdU Cell Proliferation ELISA Kit (colorimetric) (Roche, Indianapolis, IN). Cell migration was calculated on a 24-well cell migration plate (2 × 10^4^ cells) for 24-72 hrs (BD Biosciences, Franklin Lakes, NJ). Target cells (2 × 10^4^ cells) were incubated for 24-72 hrs using the BD BioCoat Matrigel invasion chambers (BD Biosciences) for invasion assays. For the 3-dimensional (3D) culture assays, anti-miR-93, mimic-miR-93 and control transfected tumor cells (2 × 10^3^ cells) were cultured on Perfecta3D Hanging Drop plates (3D Biomatrix Inc., Ann Arbor, MI) for 10 days.

### Flow cytometry

Propidium iodide (BD Bioscience) and the fluorescein isothiocyanate-conjugated (FITC) anti-human Annexin V Apoptosis Detection Kit I (BD Pharmingen) were used to characterize cells. Labeled cells (1 × 10^6^) were analyzed using the BD FACS Aria II Cell Sorter System (BD Biosciences), followed by data analysis using the Diva program (BD Biosciences).

### miR and mRNA expression analysis

The details of the quantitative polymerase chain reaction (qPCR) have been described previously [34]. Primers (miR-93, 181a, and 125a-5p) were acquired from the PerfeCTa miRNA Assays (Quanta Biosciences, Gaithersburg, MD). Subsequent detection of qPCR was performed using the PerfeCTa SYBR Green SuperMix (Quanta Biosciences, Gaithersburg, MD) with the Bio-Rad CFX96 Real Time PCR Detection System (BioRad, Hercules, CA) in a blinded fashion. Minimum Information for publication of Quantitative Real-Time PCR Experiments (MIQE) guidelines were used for the assessment and presentation of results [35]. All figures show the mean and SD of the experiments. Quantification was performed using the −ΔCq method with miR-181a as the reference gene and was verified by normal liver specimens and normal hepatocyte cell lines (Supplemental Figure 10 A-B). Gene-specific oligonucleotide primers were designed for PCR. The following primers were used: the *α-fetoprotein* (*AFP*) forward primer, 5′-ATTGTCTGCAGG ATGGGGAA-3′, and reverse primer, 5′-GTTCCAGCGTGGTCAGTTTG-3′. Quantitative expression of AFP (−ΔCq) is referenced by the human *β2-microglobrin (β2-MG)* forward primer, 5′-TGTCACAGCCCAAGATAG-3′, and reverse primer 5′-CAAGCAGCA GAATTTGGAA-3′.

### Immunohistochemistry (IHC)

IHC procedures were completed as previously described [36]. For antigen retrieval, slides were incubated in 1x citrate buffer (pH 6.0, DBS, Pleasanton, CA) at 100°C in a water bath for 10 min. After peroxidase and protein blocking and treatments, the tissue sections were incubated with the anti-human c-Met antibody (Ab) (2μg/mL, rabbit polyclonal Ab, Santa Cluz Biotechnology, Dallas, TX) at room temperature in a humid chamber for 1 hr. The sections were then visualized using the CSAII kit (Dako, Carpinteria, CA) and Vector VIP Peroxidase (HRP) Substrate Kit (Vector Laboratories, Burlingame, CA) according to the manufacturers' instructions. The sections were counterstained with hematoxylin. Photographs of the IHC staining were taken for analysis using the Nikon Eclipse Ti microscope and NIS elements software (Nikon, Melville, NY). IHC staining intensity values of specimens on slides without the primary Ab were subtracted from the corresponding specimen with the primary Ab to exclude the influence of pigmentation and background staining.

### Western blotting

Cell lysate was prepared for western blotting as previously described [37]. Protein concentrations were determined using the Pierce BCA Assay (Thermo Scientific, Waltham, MA). Membranes were immunoblotted overnight with the following primary Abs: rabbit anti-human PTEN Ab, rabbit anti-human p85 Ab, rabbit anti-human phospho-p85 Ab, rabbit anti-human p110-alfa Ab, rabbit anti-human p110-beta Ab, rabbit anti-human p110 gamma Ab (1:1000, Cell Signaling, Danvers, MA), rabbit anti-human c-Met Ab (1:250, Life technologies), rabbit anti-human phosphor-c-Met Ab (p-c-Met), rabbit anti-human total-Akt Ab (1:500, Cell Signaling), rabbit anti-human phospho-Akt (p-Akt) Ab (Ser473, 1:100, Cell Signaling), mouse anti-human CDKN1A Ab (1:200, BD Pharmingen) and mouse anti-human β-actin Ab (loading control; 1:5000, Cell Signaling). After immunoblotting, the membranes were washed with 1X TBS containing 0.1% Tween-20, followed by a 1-hr incubation with horseradish peroxidase-conjugated rabbit anti-mouse Ab (1:5000, Santa Cruz Biotechnology) and goat anti-rabbit Ab (1:5000, Santa Cruz Biotechnology). Immunoreactive bands were visualized with the Super Signal West Dura Extended Substrate kit (Thermo Scientific) and the densities of the protein bands were quantified by the Alpha-Ease FCTM software (version 3.1.2, Alpha Innotech, San Leandro, CA).

### RNA sequencing

Total RNA was isolated and mRNA was sequenced from cell lines transfected with anti-miR-93 or control miR. Starting with 2.5 μg of high quality (RIN ≥ 8.0) and high purity (OD 260/280 – 1.8-2.0) total RNA, mRNA libraries were constructed using the Illumina TruSeq RNA Sample Preparation Kit v2 following standard procedures (Illumina Inc., San Diego, CA). A coverage of over 30 million paired-end reads was obtained for each sample on the Illumina HiSeq 2500 (Illumina Inc., San Diego, CA). Reads were mapped using the TopHat2 sequence alignment program [http://genomebiology.com/content/pdf/gb-2013-14-4-r36.pdf] and expression was called using the Cufflinks software program [http://www.nature.com/nbt/journal/v28/n5/full/nbt.1621.html]. Because classical statistical tests can yield many false-positive and false-negative results when only three replicates for each case are used, fold-change was used to screen for differentially expressed genes between the anti-miR-93 and control transfected cells, and *p*-values were generated for the genes of interest.

### Biostatistical analysis

The Student's t and Wilcoxon tests for continuous variables and the χ^2^ and Fisher's exact tests for categorical variables were performed. Survival curves were generated using the Kaplan-Meier method and were compared using a log-rank test. Univariate and multivariate survival analyses were performed using the Cox's proportional hazards (Cox PH) regression model. All statistical analyses used JMP (JMP version 8.01, SAS Institute, Cary, NC) or statistical scripting language R (http;//www.r-project.org/). *P*-values ≤0.05 (two-sided) were considered statistically significant. This biomarker prognostic study complied with the REporting recommendations for tumor MARKer prognostic studies (REMARK) Guidelines [38].

## SUPPLEMENTARY MATERIAL FIGURES


