# Supplementary material for: MicroRNA-93 activates c-Met/PI3K/Akt pathway activity in hepatocellular carcinoma by directly inhibiting PTEN and CDKN1A
Source: Oncotarget. 2014 Dec 26;6(5):3211–24. doi: 10.18632/oncotarget.3085 (PMC4413648; doi:10.18632/oncotarget.3085)
Supplement: Supplementary file 1 [file oncotarget-06-3211-s001.pdf]

MicroRNA-93 activates c-Met/PI3K/Akt pathway activity in hepatocellular carcinoma by directly inhibiting PTEN and CDKN1A

Supplementary Material

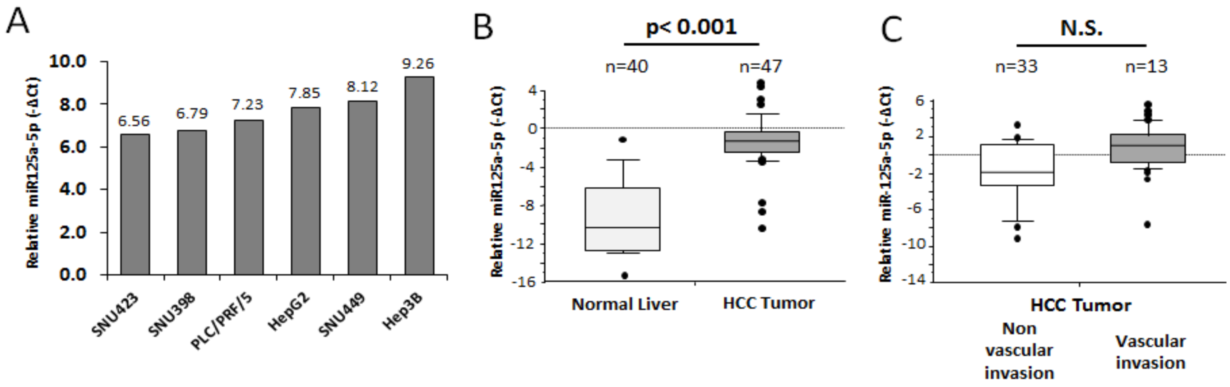

Supplemental Figure 1: Expression of miR-125a-5p *in vitro* and *in vivo*. (A) Expression of miR-125a-5p in HCC cell lines. **(B)** Expression of miR-125a-5p in HCC specimens (n=47) and adjacent non-HCC specimens (n=40). **(C)** Expression of miR-125a-5p was not significantly different in 13 HCC specimens with vascular invasion versus 33 HCC specimens without vascular invasion.

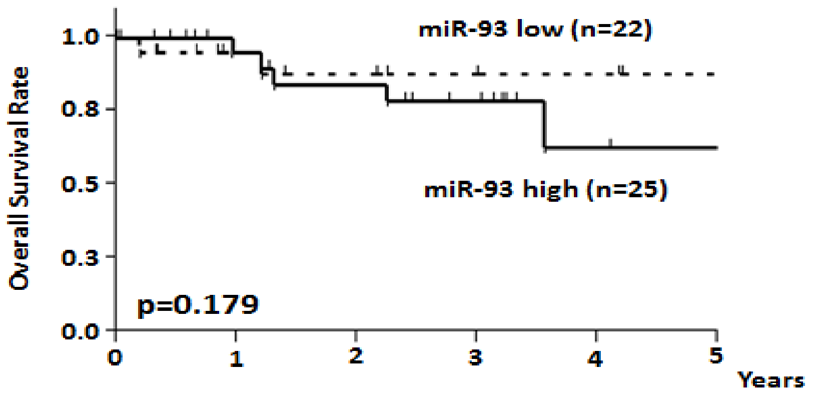

Supplemental Figure 2: Comparison of miR-93 high expression group and low expression group. Overall survival curve using Kaplan Meier analysis (Log-rank test, p=0.179).

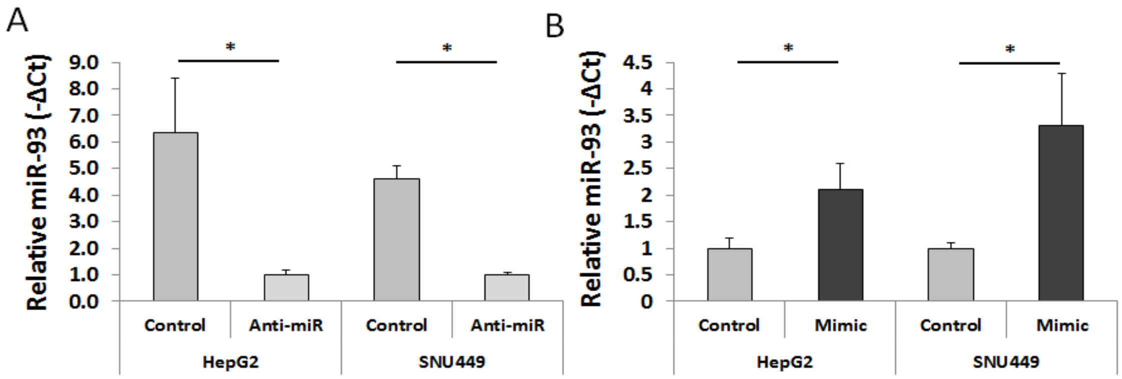

Supplemental Figure 3: Knockdown and overexpression of miR-93 expression in HCC cells

Two HCC cell lines show a decreased expression of miR-93 after transfection for 48 hrs with anti-miR-93 versus control oligonucleotides. (**A**) Relative decrease was 6.4-fold for HepG2 and 4.3-fold for SNU449 using anti-miR-93. (**B**) Relative increase was 3.3-fold for SNU449 and 2.1-fold for HepG2 using mimic-miR-93. * $p < 0.05$.

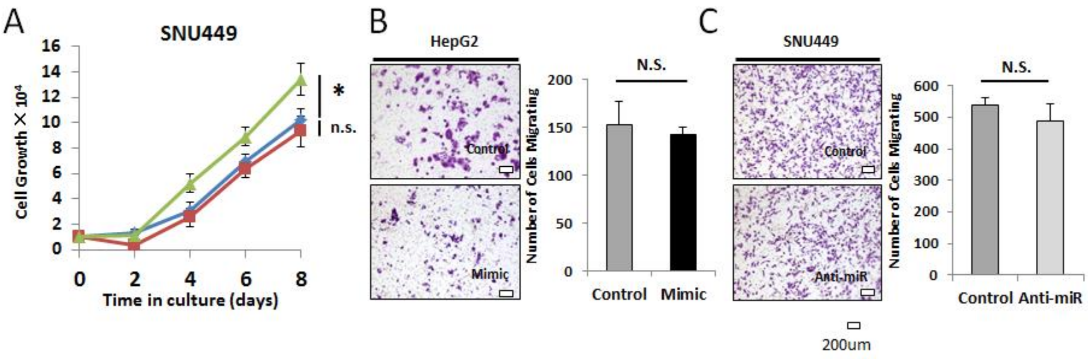

Supplemental Figure 4: Anti-miR and mimic-miR transfected cells in cell proliferation, migration, and invasion assays. **(A)** Proliferation of anti-miR-93-transfected (red), mimic-miR-transfected (green), and control-transfected SNU449 cells (blue) for 8 days. **(B)** Cell migration had no difference between mimic-miR-transfected and control-transfected HepG2 cells after 48 hrs. Scale bar = 200µm. (**C**) Cell migration had no difference between anti-miR-transfected and control-transfected SNU449 cells after 48 hrs. Scale bar = 200µm. * $p < 0.05$.

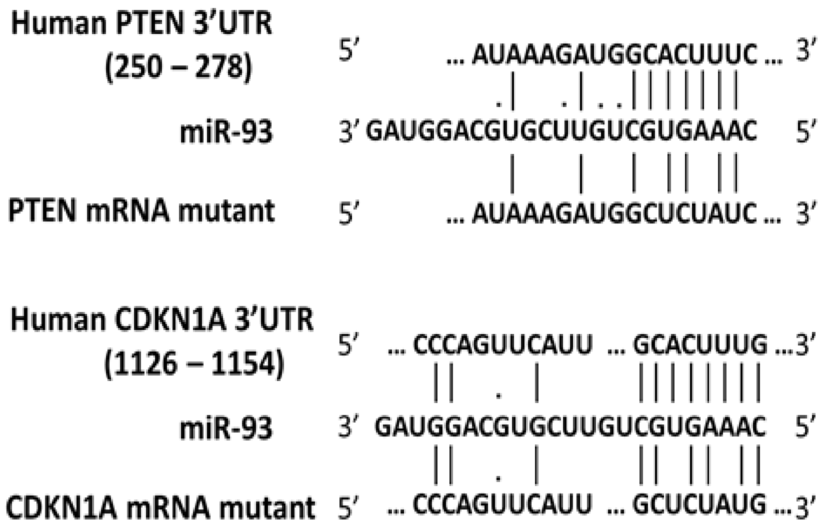

Supplemental Figure 5: The sequences of mutated *PTEN* and *CDKN1A* 3'UTR

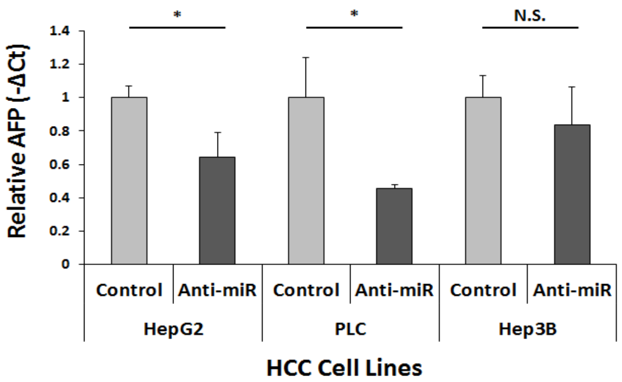

Supplemental Figure 6: Transfection of HCC cells with anti-miR-93 decreased cell expression of AFP. We performed qRT-PCR to confirm the expression of AFP as assessed by RNA sequencing data. HepG2 and PLC decreased AFP mRNA expression 48 hrs after transfection with anti-miR-93 versus control oligonucleotides. Hep3B cells also decreased AFP expression, but not statistically significant. -ΔCq referenced by β2MG. * $p < 0.05$.

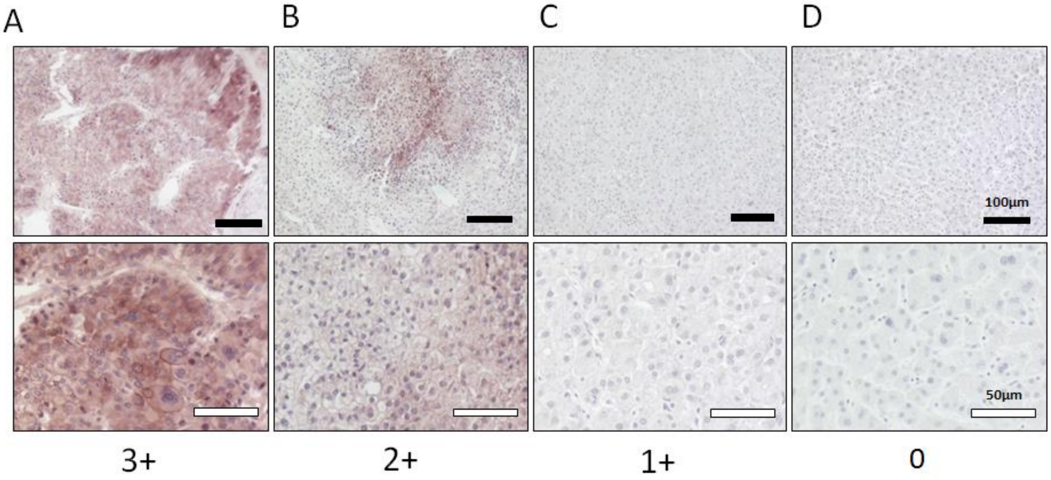

Supplemental Figure 7: c-Met IHC intensity in PEAT. Representative photograph of 3-µm PEAT liver tissue sections stained for c-Met. Four levels of IHC staining were observed in HCC PEAT: 3+ **(A**; strong**)**, 2+ **(B**; intermediate**)**, and 1+ **(C**; weak**)**. (**D**) In contrast, non-HCC liver specimens showed either weak or negative c-Met staining. Upper scale bar = 100µm, lower scale bar = 50µm.

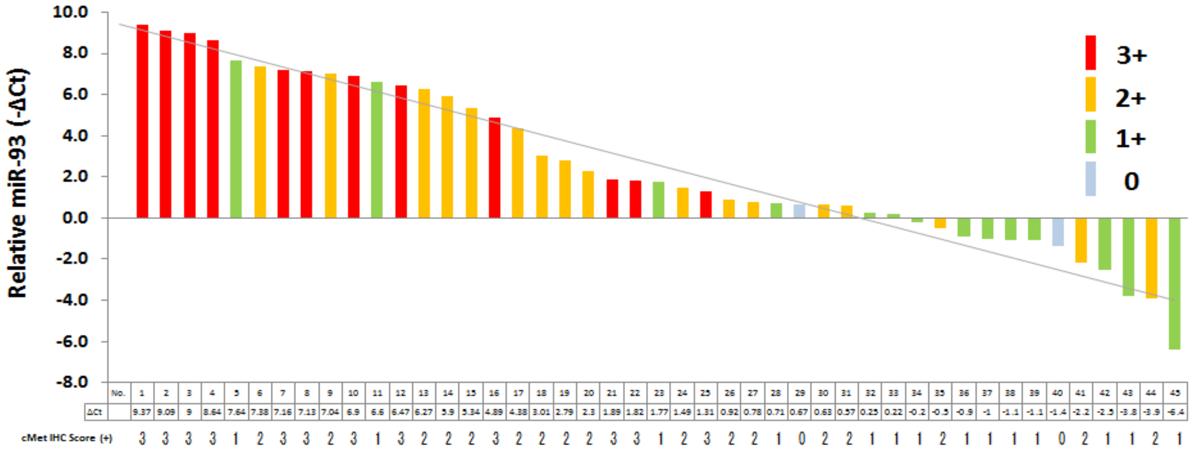

Supplemental Figure 8: miR-93 expression correlates with c-Met IHC staining intensity. The relative expression of miR-93 in HCC specimens was linearly correlated with the level of c-Met staining (staining scores: 3+, red; 2+, yellow; 1+, green; 0, blue). Expression of miR-93 was significantly higher in c-Met positive HCC specimens (Fisher exact test).

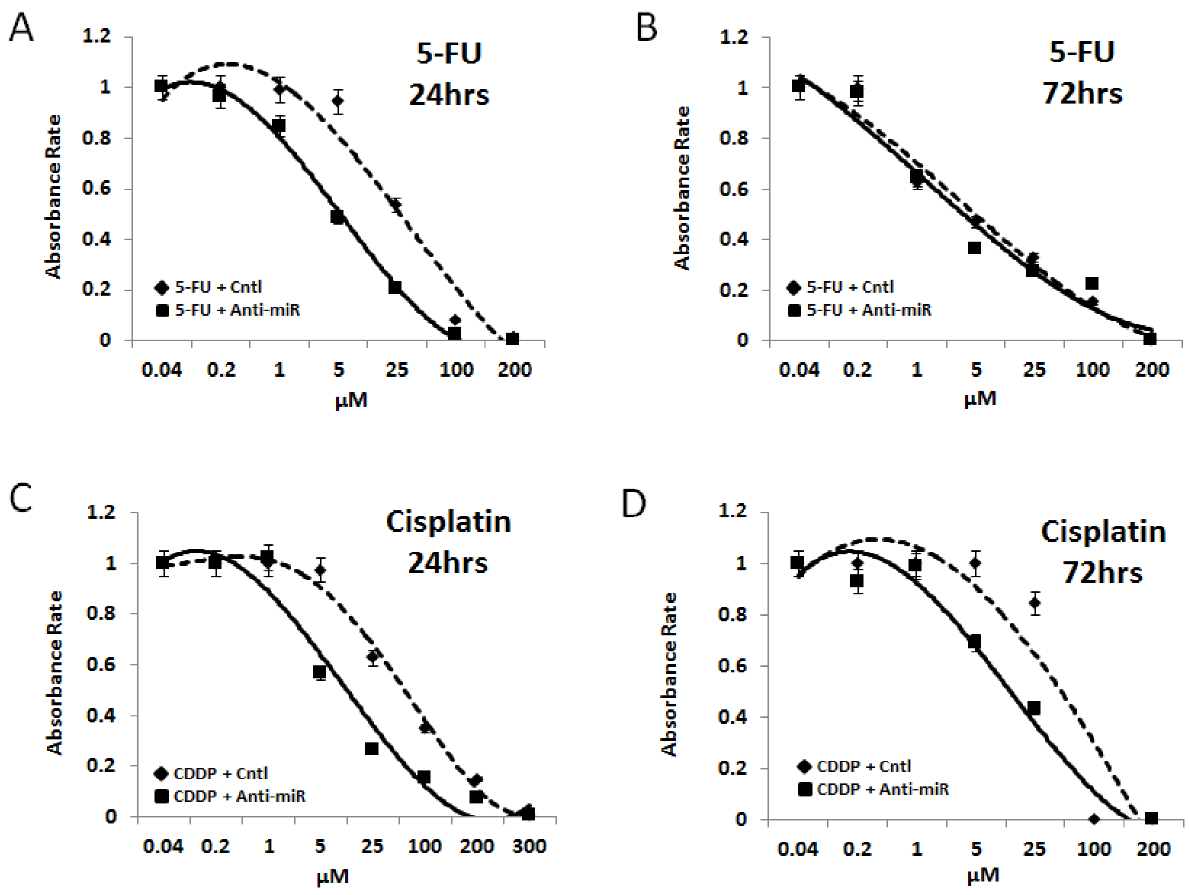

Supplemental Figure 9: Knockdown of miR-93 enhanced drug-sensitivity of transfected cells to conventional chemotherapy. Growth inhibition curves for high miR-93 expressing HCC cell lines in the presence of conventional chemotherapy for 24-72 hrs. **(A)** anti-miR-93-transfected, and control-transfected HepG2 cells exposed to 5-FU for 24 hrs. **(B)** to 5-FU for 72 hrs. **(C)** to cisplatin for 24 hrs. **(D)** to cisplatin for 72 hrs.

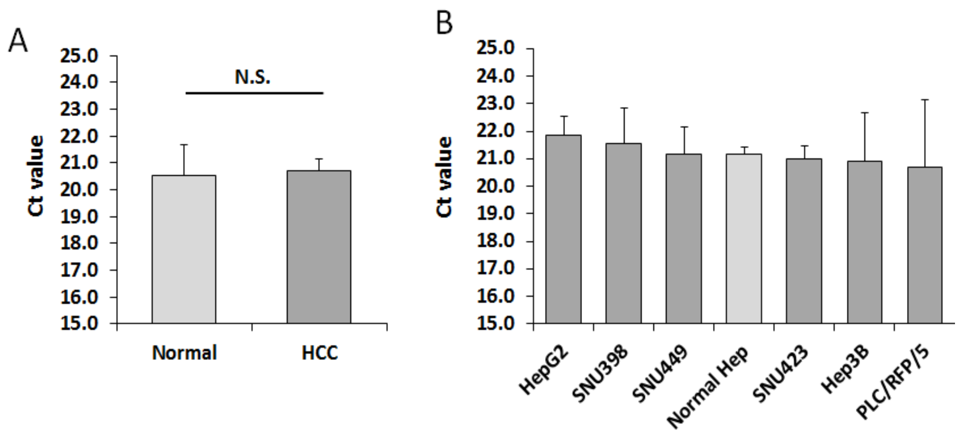

Supplemental Figure 10: qPCR expression of miR-181a *in vivo* and *in vitro*. (A) Comparison of miR-181a in clinical samples (normal liver specimens, n=10; and HCC specimens, n=22). **(B)** Expression of miR-181a in 6 HCC cell lines and normal hepatocyte cells.
